# Supplementary material for: Loss of p53 Sensitizes Cells to Palmitic Acid-Induced Apoptosis by Reactive Oxygen Species Accumulation
Source: Int J Mol Sci. 2019 Dec 12;20(24):6268. doi: 10.3390/ijms20246268 (PMC6941153; doi:10.3390/ijms20246268)
Supplement: Supplementary file 1 [file ijms-20-06268-s001.pdf]

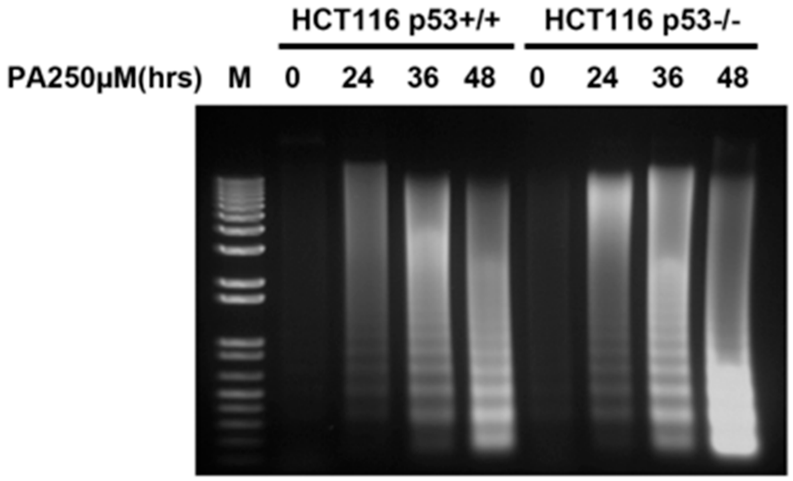

Figure S1. Apoptotic cell death was also dependent on PA treatment time in HCT116 $p53^{+/+}$ and $p53^{-/-}$ cell lines. HCT116 $p53^{+/+}$ and $p53^{-/-}$ cells were treated with 250 μM palmitic acid or BSA as a control for the indicated times. Cells were harvested, and apoptotic small fragmental DNA was extracted using lock gel.

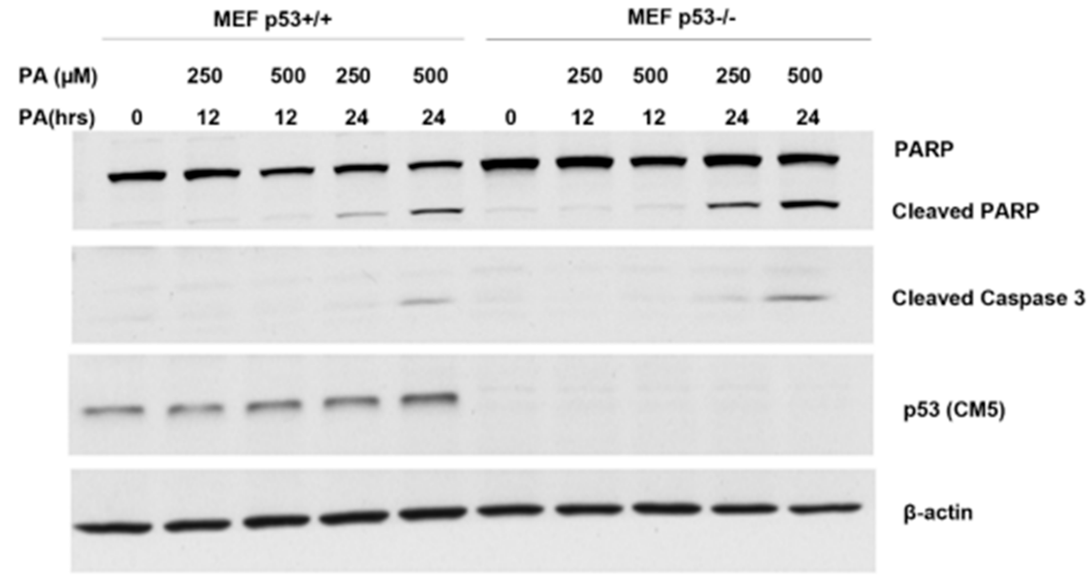

Figure S2. Palmitic acid induces MEF cell apoptosis and activates p53 in time and dosage dependent manner. Primary MEF cells were treated with palmitic acid for the indicated time and dosage. 40 μg of total protein extract was resolved on SDS-PAGE. p53, cleaved caspase-3, PARP, and cleaved PARP were detected by western blotting. β-actin was used as a loading control.
